# UPLC/MS MS data of testosterone metabolites in human and zebrafish liver microsomes and whole zebrafish larval microsomes

**DOI:** 10.1016/j.dib.2017.11.079

**Published:** 2017-12-06

**Authors:** Moayad Saad, Sebastiaan Bijttebier, An Matheeussen, Evy Verbueken, Casper Pype, Christophe Casteleyn, Chris Van Ginneken, Louis Maes, Paul Cos, Steven Van Cruchten

**Affiliations:** a Applied Veterinary Morphology, Department of Veterinary Sciences, Faculty of Pharmaceutical, Biomedical and Veterinary Sciences, University of Antwerp, Universiteitsplein 1, B-2610 Wilrijk, Belgium; b Natural Products & Food Research and Analysis (NatuRA), University of Antwerp, Universiteitsplein 1, B-2610 Wilrijk, Belgium; c Flemish Institute for Technological Research (VITO), Business Unit Separation and Conversion Technology (SCT), Boeretang 200, 2400 Mol, Belgium; d Laboratory of Microbiology, Parasitology and Hygiene (LMPH), Faculty of Pharmaceutical, Biomedical and Veterinary Sciences, University of Antwerp, Universiteitsplein 1, B-2610 Wilrijk, Belgium

## Abstract

This article represents data regarding a study published in Toxicology in vitro entitled “ in vitro CYP-mediated drug metabolism in the zebrafish (embryo) using human reference compounds” (Saad et al., 2017) [1]. Data were acquired with ultra-performance liquid chromatography – accurate mass mass spectrometry (UPLC-amMS). A full spectrum scan was conducted for the testosterone (TST) metabolites from the microsomal stability assay in zebrafish and humans. The microsomal proteins were extracted from adult zebrafish male (MLM) and female (FLM) livers, whole body homogenates of 96 h post fertilization larvae (EM) and a pool of human liver microsomes from 50 donors (HLM). Data are expressed as the abundance from the extracted ion chromatogram of the metabolites.

**Specifications Table**

**Table:** 

Subject area	*Biology*
More specific subject area	*Toxicology*
Type of data	*Table, text file, figure*
How data was acquired	*Mass spectroscopy*
Data format	*Analyzed*
Experimental factors	*Microsomal proteins were extracted from livers of 10 male and 10 female zebrafish, 1500 whole body larvae and 50 human donors livers.*
Experimental features	*Microsomal stability assay was performed by incubation of 1 mg microsomal proteins with 40 µM testosterone for two hours at 28.5 °C. The proteins were denatured using 70% acetonitrile, centrifuged at 10 000g for 10 minutes and diluted in water till 30% acetonitrile.*
Data source location	*Flemish Institute for Technological Research (VITO), Business Unit Separation and Conversion Technology (SCT), Boeretang 200, 2400 Mol, Belgium*
Data accessibility	*Data are presented in this article*

**Value of the data**

• Our data showed significant differences in metabolite profile of TST between man and zebrafish.
• These differences can result in different pharmacodynamic or toxicological effects depending on the pharmacological characteristics of the metabolites.
• In contrast to previously published articles [2], [3], [4], the main metabolite of TST in humans, i.e. hydroxytestosterone, was not detected in zebrafish
• Low concentrations or absence of metabolites in larvae at 96 h post fertilization (hpf) indicate a low metabolic capacity at this early developmental stage.
• The detected differences can influence the use of zebrafish embryos and larvae as alternative model in toxicity testing.

## 1 Data

• *Isomeric metabolites in FLM and MLM:*
  Two isomeric metabolites were detected at *m*/*z* 275.23, corresponding to the molecular formula of C19H31O ([M+H]+). No differences were found in the fragmentation spectra that were detected at *m*/*z* 275.23691. A very intense signal was observed at *m*/*z* 257.22 ([M+H-H2O]+). Minor product ions were formed at *m*/*z* 109 and 123 for these metabolites.
  Six other low abundant isomeric metabolites were detected at *m*/*z* 291.23, corresponding to C19H31O2 ([M+H]+). The relative abundance of these isomers differed significantly between FLM and MLM samples.
  Two very low abundant peaks were detected at *m*/*z* 331.22, corresponding to C19H32O3Na ([M+Na]+), which indicates a gain of one oxygen and four hydrogen atoms with respect to TST. The highest abundance of these isomers was found in FLM samples.
  Very low abundant peaks were detected in negative ionization mode, namely three at *m*/*z* 467.26, two at *m*/*z* 499.29, one at *m*/*z* 497.27 corresponding to C25H39O8 and C26H43O9, C18H40O9, respectively (Table 1).

**Table 1:** Abundance of different metabolites of TST in zebrafish female liver microsomal proteins (FLM), zebrafish male liver microsomal proteins (MLM), human liver microsomal proteins (HLM) and microsomal proteins of whole body homogenates of 96 h post fertilization zebrafish larvae (EM). (*) positive ionization mode, (**) negative ionization mode.

*m/z*	Most probable molecular formula	Abundance							
		HLM		FLM		MLM		EM	
		Time: 0	Time: 120	Time: 0	Time: 120	Time: 0	Time: 120	Time: 0	Time: 120
*275.23	C_19_H_30_O	0	1.29E+04	0	5.37E+05	0	7.54E+05	0	4.69E+04
*257.22	C_19_H_28_	0	2.87E+04	0	1.28E+06	0	2.25E+06	0	5.45E+04
*291.23	C_19_H_30_O_2_	0	2.56E+04	0	2.92E+05	0	1.24E+05	0	6.01E+03
*331.22	C_19_H_32_O_3_	0	2.11E+04	0	1.74E+05	0	1.82E+04	0	4.56E+03
*305.21	C_19_H_28_O_3_	1.41E+05	1.47E+07	3.36E+04	3.01E+04	3.74E+04	3.74E+04	3.25E+04	8.98E+04
**467.26	C_25_ H_38_ O_8_	0	0	0	2.41E+05	0	2.51E+04	0	0
**497.27	C_18_ H_40_ O_9_	0	0	0	2.74E+04	0	5.89E+05	0	3.65E+04
**499.29	C_26_H_42_ O_9_	0	0	0	1.85E+05	0	4.53E+05	0	9.28E+03
• *6β-hydroxytestosterone:*
  Nine metabolic isomers at *m*/*z* 305.21 corresponding to C19H29O3 ([M+H]+) were detected in positive ion mode only in HLM. The most intense isomer (metabolite 4) was identified as 6β-hydroxytestosterone (6β-OHTST) by comparison with an analytical standard. As expected, only very low abundant peaks were detected at *m*/*z* 97 and 109 for the 6β-OHTST isomer (metabolite 4) [5] (Fig. 1).

**Fig. 1:**
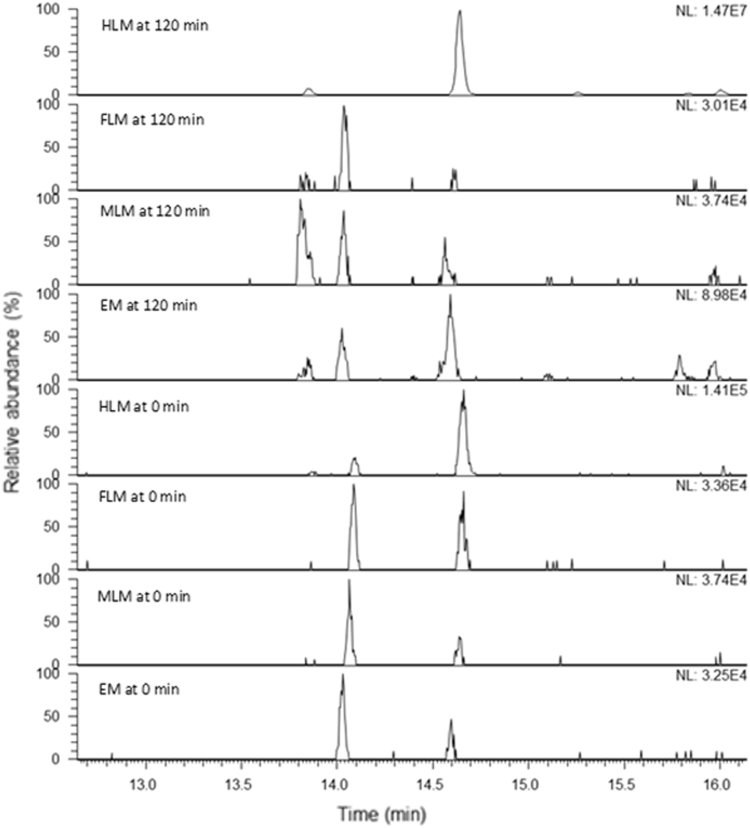
Extracted ion chromatogram of the metabolites at *m*/*z* 305.2 (hydroxytestosterone) in zebrafish female liver microsomal proteins (FLM), zebrafish male liver microsomal proteins (MLM), human liver microsomal proteins (HLM) and microsomal proteins of whole body homogenates of 96 h post fertilization zebrafish larvae (EM) at time 0 and 120 min in the microsomal stability assay of TST.
• *Very low or no significant peaks were detected in EM.*

## 2 Experimental design, materials and methods

Microsomal protein stability assay and sample extraction were conducted as stated in [1]. The extracts were analyzed as described previous [6]. Briefly, a volume of 5 µL of extract was injected with a CTC PAL™ autosampler (CTC Analytics) on a Waters Acquity UPLC BEH SHIELD RP18 column (3.0 mm×150 mm, 1.7 µm) and thermostatically (40 °C) eluted with an Accela™ quaternary solvent manager and a ‘Hot Pocket’ column oven (Thermo Fisher Scientific). The mobile phase solvents consisted of water + 0.1% formic acid (A) and acetonitrile + 0.1% formic acid (B), and a gradient as described in Table 2. For detection, an accurate mass spectrometer (amMS, Q Exactive™; Thermo Fisher Scientific) was used with heated electrospray ionization (HESI). During a first analysis, full scan data were acquired using polarity switching with an *m/z* range of 120–1800 and resolving power set at 70,000 at full width at half maximum (FWHM). Spray voltage was set at ± 2.5 kV, sheath gas and auxiliary gas at 47 and 15 (adimensional) and capillary temperature at 350 °C. Fragmentation data were also recorded using higher-energy collisional dissociation (HCD) and data dependent fragmentation (ddMS^2^) in positive and negative ionization mode (one analysis per mode) to obtain additional structural information (resolving power set at 17,500 FWHM, stepped collision energy 10, 30, 50 V, isolation window: 4 mass/charge (*m*/*z*), top 10 of most abundant ions selected for fragmentation). If fragmentation was not triggered due to low abundance of metabolites, targeted MS^2^ was carried out in an effort to get diagnostic fragmentation spectra.

**Table 2:** The gradient of the mobile phase 30% acetonitrile for the identification of testosterone metabolites. Solvent A: water + 0.1% formic acid, solvent B: acetonitrile + 0.1% formic acid.

**Time (minute)**	**%A**	**%B**
0	99	1
9.91	74	26
18.51	35	65
18.76	0	100
20.76	0	100
20.88	99	1
23.00	99	1

SIEVE software for differential analysis (Thermo Fisher Scientific) and Xcalibur software (Thermo Fisher Scientific) were used for processing the raw data. Comparison of TST samples before and after metabolism and blank samples (no addition of substrate) allowed to locate the metabolites that were formed. Structures were assigned to unknown peaks only when both the *m/z*-ratios and molecular formulae of the precursor and product ions were in agreement.
